# Falls Reduction and Exercise Training in an Assisted Living Population

**DOI:** 10.1155/2015/957598

**Published:** 2015-08-05

**Authors:** Kimberly J. Alvarez, Shannen Kirchner, Serena Chu, Sarah Smith, Wendy Winnick-Baskin, Thelma J. Mielenz

**Affiliations:** ^1^Department of Epidemiology, Mailman School of Public Health, Columbia University, 722 West 168th Street, New York, NY 10032, USA; ^2^College of Physicians and Surgeons, Columbia University, New York, NY 10032, USA; ^3^Atria Senior Living, Louisville, KY 40202, USA

## Abstract

Multicomponent exercise programs are currently an efficacious fall prevention strategy among community dwelling older adults although research documents differential falls susceptibility among frail older adults. This study aimed to examine the association between the Boston FICSIT (Frailty and Injuries: Cooperative Studies of Intervention Techniques) exercise program (the original exercise program to demonstrate that nursing home residents can increase strength) and falls incidents in an assisted living community. A descriptive cross-sectional study matched exercise charts for frequency and duration of training with number of reported fall incidents. Among 39 participants, 33% (*n* = 13) reported a fall incident. Adults without a fall history reported more time in aerobic (26.30 versus 20.00, *P* value = 0.71) and strength (1.50 versus 0.50, *P* value = 0.01) training sessions compared to those with a fall history. Multivariate models adjusting for covariates illustrated a significant protective association between strength training and fall incidents (OR = 0.25; 95% CI = 0.07, 0.85). In this cross-sectional study, this progressive resistance exercise training program into an assisted living population was associated with a decrease in the number of fall incidents.

## 1. Introduction

In the US, 30% of older adults experience at least one fall annually, with a particularly higher rate (50%) over the age of 80 [1]. Falls not only are the leading cause of injury death, but also are the most common cause of nonfatal injuries and lead to chronic pain and disability [2]. An estimated $30 billion dollars was spent in 2010 on inpatient hospitalizations and outpatient medical costs related to falls [3]. Falls create an enormous economic burden and effective falls prevention will reduce health care costs and increase quality of life in older age [4].

The US Preventive Services Task Force states that multifactorial risk assessments offer a small benefit for falls prevention in community dwelling (CD) older adults [5]. Some risk factors can be modified using exercise (e.g., impaired balance and gait), whereas others require different intervention approaches (e.g., poor vision) [6]. On the other hand, exercise training programs for CD adults have considerable benefit and can be used as a stand-alone falls prevention intervention or as a component of a multifaceted program. Prior studies have established the fact that exercise interventions for falls reduction and injury prevention are effective in CD older adults [5–11]. However, high interindividual variability and differential falls susceptibility in CD versus frail older adults suggest falls prevention programs designed for CD older adults may not be translatable to more frail older adults in assisted living (AL) and nursing homes. AL communities are the fastest-growing noninstitutional long-term care alternative in the US for older adults [12]. As a result, AL communities are an extraordinarily diverse shelter and care alternative, and very frail older persons with serious chronic health conditions can be found in this setting [12]. Limited research has examined associations between exercise programs and falls among AL populations [13].

This exercise program was designed by exercise physiologists in the 1980s and 1990s from Tufts University School of Nutrition and Center for Healthy Aging (i.e., William J. Evans, Ph.D., and Miriam E. Nelson, Ph.D.). The aim of the program was to reverse bone loss and improve energy and balance among older adults by progressive resistance exercise training [14]. This exercise program laid the foundation for current evidence-based CD programs. Moreover, it was found to be efficacious by a randomized controlled trial conducted by Fiatarone and colleagues using data from the Boston Frailty and Injuries: Cooperative Studies of Intervention Techniques (FICSIT) study [15]. Results from the trial demonstrated that it was both feasible and effective in counteracting muscle weakness and physical frailty among nursing home residents [14, 15]. Progressive resistance exercise three days a week for ten weeks significantly improved the results of all muscle-strength tests. Specifically, among those who participated in exercise training, gait velocity increased by 11.8% and overall muscle strength increased by 111.3%, compared to nonexercisers [15]. While the exercise program was successful among frail adults in a nursing home, it is not clear whether this program would be efficacious among AL populations to reduce falls.

The goal of the present study was to evaluate the association of the aerobic and strength training exercise program, adopted from Fiatarone et al., to reduce falls incidents and falls-related injuries in an AL population [15].

## 2. Materials and Methods

Participants for this five-year period prevalence cross-sectional study (2007–2011) were ascertained from one community in a privately owned, for-profit senior housing company that provides independent living, assisted living, and memory care services. Inclusion criteria for participation in the exercise program included (1) being current community resident, (2) attending a meeting with the community activity director, and (3) having signed form submitted by participant's physician. The Columbia University Medical Center Institutional Review Board approved this retrospective study.

### 2.1. Exercise Intervention

Dr. Miriam E. Nelson, the Exercise Physiologist from Tufts, came to the AL communities in the late 1990s and helped to translate the Boston FICSIT program into several communities [15]. Exercise program guidelines advised AL residents to incorporate aerobic and strength training into their regular exercise routine three times a week using equipment available in the community's fitness center. Strength training was performed on Keiser Air Resistance Equipment, with supervision, and included shoulder press, seated row, leg extension, leg curl, and leg press machines. Aerobic training was performed on NuStep machines or treadmills. Residents also had the option of participating in chair exercise classes held six days a week for one hour; these sessions included stretching, hand weights, and resistance bands. Activity directors supervising the program assisted participants in the fitness center and followed up with those who failed to attend regular sessions.

The staff member on duty in the fitness center set up the equipment and recorded mode, frequency, and duration of each activity in participants' individual exercise chart. For strength training, staff documented exercise, machine resistance, and number of repetitions and sets. For aerobic training, time, speed, and resistance were recorded. The exercise chart remained with the participant throughout the program.

### 2.2. Falls Assessment

A fall was defined as unintentionally coming to rest on the ground, floor, or any other lower level [16]. Staff recorded the fall incident using a standard incident report form. Additional information collected about the fall incident included date, injury, location, cause (as stated by participant), reason for fall, and history of falls. Fall incident report data was merged with a participants' other study information.

### 2.3. Exercise Dose and Participation Assessment

Two primary measures of exercise were retrospectively obtained from exercise charts: aerobic training in minutes and number of strength training sessions. From these chart values, three variables were calculated: years of participation, number of aerobic and strength sessions attended each week, and minutes of participation in each aerobic session.

### 2.4. Statistical Analysis

Statistical analyses were performed using SAS Version 9.2. Descriptive analyses were used to report characteristics of the study population by falls history, including age, gender, and participation in the exercise program. Differences in participation history between those with a fall history and those without it were tested using *t*-tests. Logistic regression models were designed to assess the association between volume of exercise participation and falls incidents, adjusting for gender and age.

## 3. Results

In the 39 individuals in this analysis, a total of 13 (33.3%) reported a fall incident during the five-year period. The mean age in years was 87.4 at the beginning of the study; 74.4% (*N* = 29) were female. The mean participation in the exercise program was 2.2 years, with mean aerobic and strength training at 197.7 minutes and 136.1 sessions, respectively. Among those with a fall record, a total of 60 incidents were reported; of those, half reported at least two or more falls. Most common reported reason for fall incident was loss of balance.

Results comparing study population characteristics according to fall status are shown in Table 1. Negative associations for fall incidents were recorded with total exercise program participation, aerobic training, and strength training. Weekly descriptors of training illustrated similar results. Those with no reported fall incidents documented more time in aerobic training minutes per week (26.30 versus 20.00, *P* value = 0.71) and strength training sessions per week (1.50 versus 0.50, *P* value = 0.01) compared to those with a reported fall incident.

Dose-dependent associations for fall incidents were seen with participation in the exercise program (Table 2). In adjusted models, aerobic and strength training were associated with decreased odds of a fall incident. Specifically, those who recorded a greater number of total strength training sessions were significantly less likely to experience a fall incident (OR = 0.25; 95% CI = 0.07, 0.85) than those with fewer total sessions.

## 4. Discussion

This study reports findings from a cross-sectional study investigating the association between the Boston FICSIT and fall incidents among older adults in one AL community. Our findings report a protective association between strength training and fall incidents; the odds of a fall incident among those who logged more time strength training were less compared to those who logged less time. More specifically, individuals with a greater number of strength training sessions per week had 75% reduced odds of a fall incident than those with fewer sessions.

Analysis of the Boston FICSIT cohort data by Fiatarone et al. reported this exercise training program significantly improved the results of all muscle-strength tests among exercise participants compared to controls [15]. Among those who participated in exercise training, gait velocity increased by 11.8% and overall muscle strength increased by 111.3%, compared to nonexercisers [15]. The present study attempts to investigate if this same exercise program is associated with falls reduction in the AL population. This study appears to provide additional evidence that progressive resistance exercise training, as first reported by Boston FICSIT exercise training study, is associated with reducing fall incidents in another frail older adult setting, the AL population [15].

Results of this study suggest that older adults who spent less time in the exercise program may be more susceptible to fall incidents than those with greater time spent. Our analysis is limited by the cross-sectional study design; caution in the interpretation of the observed associations must be taken into account. A second limitation was the inability to control for baseline health among participants and lack of screening for additional activity outside the prescribed exercise program. Third, incident reports, while standardized and structured, were used to enumerate the dependent variables and are likely to capture only falls requiring assistance from the facility staff. Therefore, fall incidents may be underreported in this population. Finally, there is no comparison group; this study assessed the association among only those who participated in the exercise program. Therefore, we do not have information about those who did not participate in the program and their fall incidents.

The findings of this study have important implications for AL populations and highlight the need for future research to identify specific exercise programs, specifically focusing on strength training protocols, to garner the best outcomes among this population. Future research should include the following topics: the amount of time spent in strength training to effectively reduce falls, the types of strength training exercises that are the most beneficial in reducing falls, and inclusion of both aerobic and strength training. This could facilitate interventions aimed at reducing falls incidents among vulnerable older adult populations.

## Figures and Tables

**Table 1:** Exercise participation in relation to fall status.

Characteristic	Fall history (*n* = 13)		No fall history (*n* = 26)		*P* for trend^*∗*^
	Mean ± SD	Range	Mean ± SD	Range	
Fitness program participation, days	588.8 ± 648.4	1–1,834	886.7 ± 977.2	1–2,954	0.1389
Aerobic training					
Total aerobic training sessions	130.7 ± 190.6	1–567	231.2 ± 267.4	0–859	0.2201
Aerobic training minutes per week	20.0 ± 15.6	0–52	26.3 ± 14.4	0–49	0.7101
Strength training					
Total strength training sessions	81.5 ± 158.6	0–513	163.4 ± 248.5	0–831	0.1065
Strength training sessions per week	0.5 ± 0.2	0–2	1.5 ± 1.6	0–7	0.0054

^*∗*^Results from *t*-test comparing means for fall history and no fall history groups.

**Table 2:** Multivariate logistic regression model of factors associated with fall history in an assisted living population.

Characteristic	Unadjusted OR (95% CI)	Adjusted OR^*∗*^ (95% CI)
Total program participation	1.00 (0.99, 1.00)	0.99 (0.99, 1.00)
Aerobic training	0.97 (0.93, 1.02)	0.97 (0.92, 1.03)
Strength training	0.42 (0.18, 0.96)	0.25 (0.07, 0.85)

^*^Adjusted for age and gender; *N* = 39.
